# Research on the motivation system and path simulation of collaborative agglomeration of Chinese culture and tourism industries based on system dynamics

**DOI:** 10.1371/journal.pone.0296963

**Published:** 2024-01-25

**Authors:** Yihan Chi, Yongheng Fang, Jiamin Liu

**Affiliations:** 1 School of Management Administration, Xi’an University of Architecture and Technology, Xi’an, China; 2 School of Public Administration, Xi’an University of Architecture and Technology, Xi’an, China; East China Normal University, CHINA

## Abstract

In the context of industrial integration, the collaborative agglomeration of the cultural and tourism industries is an important way to promote their integrated development and achieve both industrial transformation and upgrading. This article first analyzes the dynamics of the cultural and tourism industries as a collaborative agglomeration. A system dynamics model is then presented which represents the perspective and reveals the mechanics of this partnership between the two industries. Finally, the authors use this model to simulate the path made possible by their collaborative agglomeration. The results show: (1) From the perspective of industrial policy, the promotion and guidance function of industrial policy elements still needs strengthening in the collaborative development of China’s cultural and tourism industries. (2) From the perspective of industrial economy, the promotion function of industrial economic factors still needs improving in the collaborative agglomeration and development of China’s cultural and tourism industries. (3) From the perspective of the joint effect of industrial policy and economy, the collaborative effect of industrial policies and economic factors is more conducive to promoting the collaborative agglomeration development of China’s cultural and tourism industries. The research in this article can provide theoretical support and policy recommendations for promoting coordinated development of China’s cultural and tourism industries and can also provide the experience needed to serve as a reference for the joint development of tourism and culture in other similar regions.

## 1. Introduction

With the continuous promotion of economic globalization and the improvement of people’s material lives, the cultural industry and tourism industry have grown into interrelated industries with strong economic potential. According to statistics from the United Nations World Tourism Organization (UNWTO), about 37% of tourism activities in the global tourism industry involve cultural factors, and 40% of cultural elements are integrated with tourism activities. Cultural tourists grow at an annual rate of 15%, indicating a significant development trend of collaborative agglomeration between the cultural and tourism industries [1]. Since Ellison and Glaeser [2] first proposed the concept of industrial collaborative agglomeration, this industrial model has gradually become an important form of development for highly correlated heterogeneous industries to expand the scale of industrial economy, optimize industrial structure, and integrate industrial resources. Since the concept of integrated development of cultural and tourism industries was introduced in China in 2009, the collaborative agglomeration development was put into preliminary theoretical practice, and was regarded as the key to achieving industrial transformation and upgrading, and the key to enhancing industrial core competitiveness. The issue of coordinated agglomeration and development of the cultural and tourism industries reached a climax when the Ministry of Culture and Tourism of China was restructured in 2018. However, the current level of collaborative agglomeration between China’s cultural and tourism industries is still in its early stages [3]. There are still such problems as incomplete driving force system and unclear development path in collaborative agglomeration. And the solutions to the two problems have become the key to the bottleneck of industrial transformation.

Industrial collaborative agglomeration, as an allocation to optimize resources, is not a simple linear substitution, but rather, driven by various factors, the resource recombination and collaboration deepening of heterogeneous industries, which thereby achieves the transition of the industrial cluster system from a multi-point structure to a network structure [4]. Early research on the driving force of industrial collaborative agglomeration mostly focused on the identification, qualitative description, and functional analysis of the driving force of industrial collaborative agglomeration. Chi et al. [3] believe that there is a strong correlation between industrial collaborative agglomeration and socio-economic development. They believe that there is a strong correlation between the socio-economic level and the industrial collaborative agglomeration in economic development regions. Moreover, their research also indicates that reducing trade barriers in the region will also lead to industrial transfer. However, with the stepwise deepening of economics and the constant development of industrial agglomeration theory, research on industrial collaborative agglomeration has gradually shifted from the identification of its driving force and the analysis of action mode to qualitative analysis of the driving factors, the action degree, and the function mechanism of industrial collaborative agglomeration. These studies further expand the research perspective in the field of industrial collaborative agglomeration. As an aggregation of the labor market, industrial clusters are believed by many scholars to be influenced by the rootedness of industries. And the factors, such as input-output correlation, labor costs, and resource endowment factors, are the decisive factors for industrial collaborative agglomeration. Thus, the three factors of the combination of cultural and tourism industries, labor costs and resource endowment can form an input-output correlation, and is established as the decisive factors behind industrial collaborative agglomeration [5]. Klein & Broadberry [6] also believe that the economic externality of industry is the generating power of industrial collaborative agglomeration, in which the input-output relationship is the decisive factor. But with the continuous development of science and technology, the impact of industrial technological innovation on industrial site selection is becoming increasingly significant, and skill endowments have become one of the important factors driving industrial collaborative agglomeration. Steijn et al. [7] also believe that with the continuous optimization of the technological environment, input-output connections and labor market convergence are no longer key determinants of heterogeneous industrial collaborative agglomeration. Instead, the knowledge spillover effect is a new focus of industries gaining increasing importance. Nevzorova [8] confirmed from a spatial perspective the driving force of technological innovation on industrial collaborative agglomeration and regional innovation development, but he believes that there is an interactive mechanism between technological innovation and industrial collaborative agglomeration. Therefore, many factors affect the collaborative agglomeration of the cultural and tourism industries, and the driving factors of industrial collaborative agglomeration have diverse and complex characteristics.

From the system theory perspective, many scholars regard industrial clusters as a complex system and believe that based on system theory, we can analyze the dynamics of industrial collaborative agglomeration more comprehensively. Scholars classify the power system of industrial collaborative agglomeration into internal power systems generated by an industrial collaborative history with deep roots and external power systems strengthened by factors such as industrial policies and external competition [9, 10]. Rullani [11] established an evolution model of industrial agglomeration dynamics system from four levels: micro, meso, macro, and meso. He believed that at the micro level, the dynamics of industrial collaborative agglomeration mainly include factors such as regional division of labor, knowledge innovation, and transactions. At the meso level, its power system includes government behavior power and market competition power; At the macro and macro levels, the driving forces of industrial collaborative agglomeration include government incentives and external competition mechanisms, respectively. Igor et al. [12] used the theory of complex systems to confirm that the financial industry cluster is a complex system, and the competitive and diffusion effects generated by the financial industry cluster can effectively accelerate that cluster’s progress. The results can also have a positive effect on the innovation and diffusion of financial products. However, the driving force of industrial collaborative agglomeration is nonlinear, characterized by dynamism, complexity, and periodicity [13]. In view of this, Luo et al. [14] analyzed the dynamic system of sustainable development of the phosphorus resource industry through a system dynamics modeling, focusing on the effect of the five subsystems upon industrial development: resources, industry, economy, environment, and society on industrial development. They believe that the industrial resource subsystem, for resource-based industries, is the key to their sustainable development. Yang et al. [15] constructed a systematic network of tourism industry based on the dynamics theory, taking the factors of consumer demands as the primary power of tourism industries agglomeration; resource endowment the endogenous power; policy environment the regulatory power; and infrastructure the impetus power. And they put forward four types of tourism industries agglomeration models: “demand driven, resource dependent, externally forced, and hybrid driven models”. From this, it can be seen that the power system of the networked urban backgrounds coupled with tourism development are relatively complex. The driving factors have diverse characteristics. Clarifying the structure and driving factors of industrial collaborative agglomeration power system is the key to optimizing, developing, and promoting a combined city and tourism system.

The literature review presented herein shows that an urban networked power system supporting the development path of tourism and industrial collaborative agglomeration is the key to developing a networked tourism and industrial collaborative agglomeration with a driving force that is not simply a linear superposition. According to the urban networked tourism and culture systems theory, industry, as a complex system, has the characteristics of nonlinear, dynamic and complex dynamics to promote its collaborative agglomeration development. Moreover, the power of industrial collaborative agglomeration not only comes from the internal factors of the system, but are also affected by the external factors of the system. However, most of the current research on the power system of industrial collaborative agglomeration only analyzes the impact of internal factors on its agglomeration development, neglecting the impact of external factors. There is also a lack of research on the development path of industrial collaborative agglomeration and a lack of quantitative research on the development path of industrial collaborative agglomerations. In addition, as emerging industries, the cultural industry and tourism industry have a strong synergistic relationship, but the research on the development path of their collaborative agglomeration is still in its early stages. Thus, this article is based on the authors’ theory of system dynamics and how it applies to the collaborative agglomeration of the culture and tourism industries. Both internal and external aspects of the industrial system are presented, which explore the development path of promoting the collaborative agglomeration of the culture and tourism industries.

The authors’ collaborative agglomeration dynamic system model was constructed by taking the Chinese culture and tourism industries as the research object. The driving factors are selected based on the collaborative agglomeration power system of culture and tourism industries. This dynamic power system is empowering for several reasons. The development path of the collaborative agglomeration of culture and tourism industries is simulated using the power system analysis model. This power system is empowering for several reasons. It provides an opportunity for countermeasures and suggestions that can further develop the agglomeration of Chinese culture and tourism industries. The culture and tourism collaborative agglomeration’s power system model expands the research perspective by providing ideas on the power of participation as members becomes stronger on the development path.

## 2. Framework of motivation system of the collaborative agglomeration of the cultural and tourism industries

### 2.1 Analysis of motivation system of the collaborative agglomeration of the culture and tourism industries

The driving force behind the system of industrial collaborative agglomeration between culture and tourism is a core issue in this study. It has a complex composition principle and some challenging elements. From the perspective of systems theory, the collaborative agglomeration of heterogeneous industries is not a simple superposition of two industries, but a new multi-level and multi factor industrial system formed on the basis of the coupling relationship between the constituent elements of the two industrial systems. Pasquale et al.’s research [16] also confirmed that industrial collaborative agglomeration is an organic combination of industrial system elements, and those elements are the driving force from which the industrial collaborative agglomeration comes from (i.e., the unbalanced gap between the internal and external parts of the two industrial systems). From a systemic perspective, the flow of underlying factors within the industrial cluster system will affect the process of industrial collaborative agglomeration development. These factors come from the fundamental elements that make up the industrial cluster, which include industrial resources, the industrial economy, and human resources. In addition, the flow of factors between the external and internal parts of the industrial system can promote or restrict the coordinated, clustered development of industries. These factors mainly come from industrial regulations required by government, markets, and other industrial entities. The regulations are not unfair. They are simply a result of industrial policies, market demand, industrial innovation resources, and socio-economic factors.

**Fig 1:**
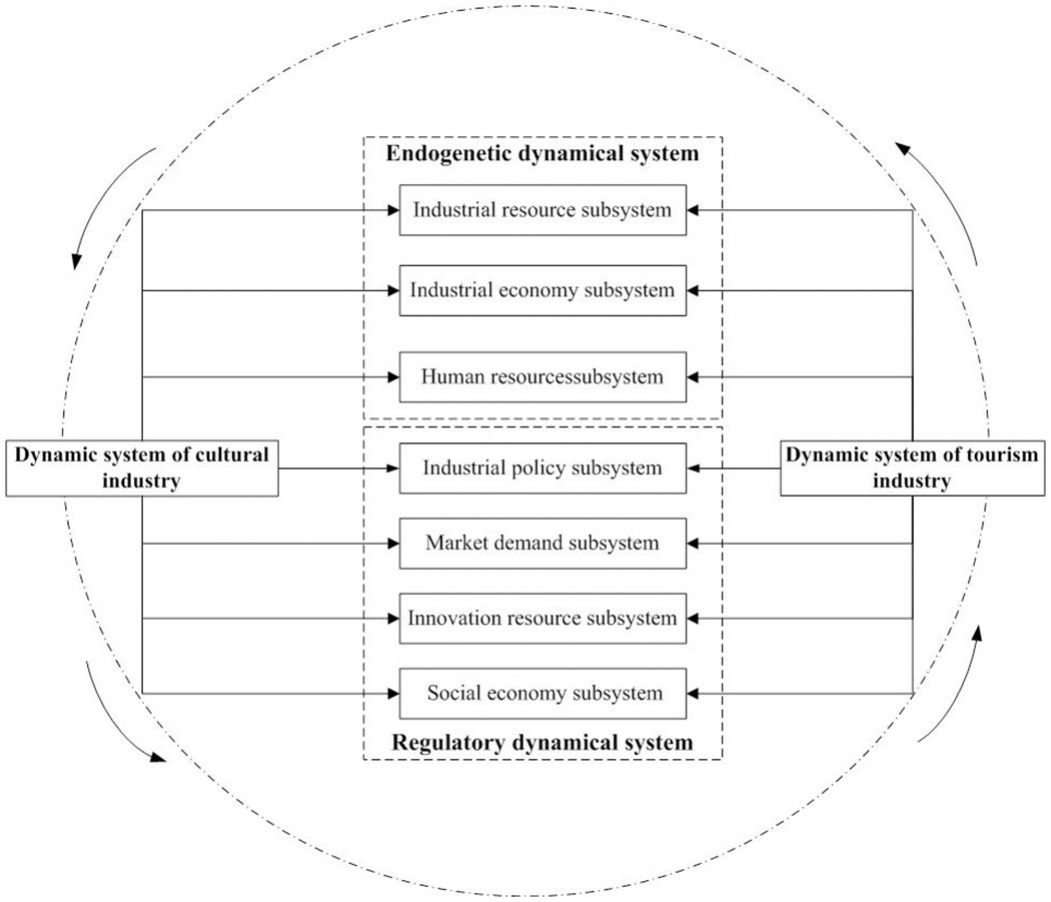
The circle of the collaborative agglomeration power system between the cultural and tourism industries. All subsystems feed their primary power systems, by providing them with the resources they need to keep the circle of communication and productivity going.

The authors’ theory of system dynamics divides the power system of the collaborative agglomeration of cultural and tourism industries into an endogenous power system and a regulatory power system. The endogenous power system has three subsystems: an industrial resource subsystem, an industrial economy subsystem, and a human resource subsystem. The regulatory power system includes an industrial policy subsystem, a market demand subsystem, an innovation resource subsystem, and a social economic subsystem. Fig 1 shows how both primary systems are empowered by the dynamics of their subsystems which are shared in a circle of empowerment.

### 2.2 Analysis of motivation mechanics of the collaborative agglomeration of the culture and tourism industries

The culture and tourism collaborative agglomeration has power systems that can drive the dynamics behind their powerful development as follows:

**(1) The endogenous power system**
  **(a) The industrial resource subsystem**: From the basic law of industrial collaborative agglomeration, the resource endowment advantage of a region is the original driving force of industrial collaborative agglomeration. Therefore, when selecting a location for an industry, it is necessary to comprehensively consider the industrial resource endowment of the selected location. Compared to traditional industries, cultural and tourism resources have such typical characteristics as geographical rootedness, monopoly, non-replicability, and scarcity. Museums and tourist attractions are the foundation of the cultural and tourism development because they are resources difficult to replicate. Therefore, the cultural and tourism industries have a higher degree of dependence on industrial resources, and regions with better cultural and tourism resource endowments are more conducive to achieving coordinated and concentrated development of the cultural and tourism industries.
  **(b) The industrial economy subsystem**: The level of industrial economy and the coordinated agglomeration and development of culture and tourism industries are a mutually promoting and interdependent process. Steijn et al. [7] believe that the level of industrial economy is a prerequisite for the coordinated agglomeration development of culture and tourism industries. The increase in industrial economic strength and income is conducive to promoting the extension of the industrial chain of culture and tourism industry, as well as promoting the coordinated agglomeration development of the two. At the same time, industrial collaborative agglomeration can promote the scale of industries and economies. From the perspective of industrial economic structure, the factors including industrial investment, industrial added value, and total industrial income are important components of industrial economy, and play important roles in promoting the collaborative agglomeration of cultural and tourism industries.
  **(c) The human resources subsystem**: According to Weber’s industrial location theory [17], human resource endowment is an important factor to consider when selecting a factory location. On the one hand, in regions with better human resource endowments have a larger stock of knowledge and higher speed of knowledge updating. The knowledge spillover effect caused by talent aggregation can reduce the cost of technological innovation. On the other hand, in regions with abundant labor resources, sufficient labor can increase industrial income, and thereby bring about economic spillover effects. In essence, the cultural industry is a knowledge-intensive trade and the tourism industry is a labor-intensive trade. Adequate human resources can improve the knowledge and economic spillover effects of the cultural and tourism industries. Therefore, regions with the advantage of abundant human resources are more conducive to the collaborative agglomeration and development of the cultural and tourism industries.
**(2) The regulatory power system**
  **(a) The industrial policy subsystem**: As an important regulatory tool for social stability and development, policies have a remarkable impact on resource allocation. Industrial policy and financial support can effectively promote the collaborative agglomeration development of industries. Mao et al.’s research [18] also confirms that the intensity of industrial policy is the supporting force in the industrial development process. For the cultural and tourism industries, there currently is a notable correlation between the two industries, but the spontaneous transfer of industries takes a long time. Therefore, the transfer and agglomeration of the two are necessarily guided by industrial policies. For the Chinese cultural and tourism industries, the transfer and agglomeration of industries require financial support, and factors like the amount of industrial financial investment and the proportion of industrial expenses to fiscal expenditures are definitely the key driving forces for their coordinated agglomeration development.
  **(b) The market demand subsystem**: Krugman’s [19] "core-edge" model confirms that a large enough market demand is a factor that must be considered when selecting industrial locations. For the cultural and tourism industries, the sufficient market consumers are the foundation for the coordinated development of the two industries. According to Maslow’s hierarchy theory of basic needs, cultural needs belong to a higher level of needs based on material needs. When people’s material lives are greatly satisfied, they will have a huge spiritual and cultural need. However, consumers’ needs are difficult to meet by single cultural product or tourism product, and developing integrated cultural and tourism products has become the key to meeting market demand. Therefore, market demand factors are important factors in the collaborative agglomeration power system of cultural and tourism industries.
  **(c) The innovation resource subsystem**: With the development of science and technology and the rise of industrial revolution, the traditional extensive economic development mode is difficult to sustain. Social and economic development has shifted from the mode of being driven by traditional production factors to that of being driven by technology. Hervas-Oliver et al. [20] point out that technological innovation can significantly change the turning point of the industrial collaborative agglomeration development, and innovative talents are the key element of industrial technological innovation. For the cultural and tourism industries, the regions with innovative resource endowments can easily form knowledge spillover effects and break through technological barriers between industries, and can promote the clustering of cultural and tourism industries in the regions with innovative resource endowments, and can eventually achieve coordinated development of cultural and tourism industries. Therefore, the innovative resources are important factors in the collaborative agglomeration power system of cultural and tourism industries.
  **(d) The social economic subsystem**: Marshall’s external economic theory [21] suggests that there is a coupling relationship between social economic and industrial development levels, and social economic factors have a strong regulatory effect on industrial development. For the cultural and tourism industries, the growth of social economy is the foundation for the development of the two industries, and it is also the key to the industrial collaboration and agglomeration. At the same time, the collaborative agglomeration of cultural and tourism industries can promote industrial transformation and upgrading, thereby achieving large-scale industrial economies and accelerating social economic development. It is thus clear that social economic development is the external driving and regulatory force for the collaborative agglomeration of cultural and tourism industries.

## 3. Case study

### 3.1 Research object

China has abundant cultural and tourism resources, and the development of cultural and tourism industries plays a crucial role in the development of China’s national economy. Current research indicates that the integration of culture and tourism industries can accelerate industrial transformation and upgrading, can speed up social economic development. And promoting industrial collaboration and agglomeration is an essential means to enhance the degree of industrial integration [22, 23].

Although the degree of collaboration and agglomeration between China’s culture and tourism industries has been continuously improving in recent years, according to the *Big Data Report on the Development of China’s Culture and Tourism Industries*, the average composite index was only 0.39 for the integrated development of culture and tourism industries in 2020, and its total output value only accounts for 8.44% of GDP. In the initial stage of the collaborative agglomeration development of Chinese culture and tourism industries, exploration and analysis of the driving force system and development path of their collaborative agglomeration are the key to promoting the agglomeration development of the two industries. At the same time, as the second largest economy, China’s coordinated development path of cultural and tourism industries also has considerable reference value for other similar regions in the world. On this account, this article selects China (excluding the Hong Kong, Macao, and Taiwan regions) as the research object, aiming at unveiling the dynamical system and the development path of the collaborative agglomeration of the cultural and tourism of industries.

### 3.2 Selection of power elements and data sources

In order to make further quantitative analyses from the systematic perspective on the collaborative agglomeration power system of China’s cultural and tourism industries, this article constructs an indicator system of the collaborative agglomeration power factors, which are based on the research of Kumar [24]、Li [25] and Yang [26]. The statistics are provided in the *Chinese Culture and Tourism Statistical Yearbook*, *China Tourism Statistical Yearbook*, and *the Chinese Culture and Related Industries Statistical Yearbook*. As shown in Table 1:

**Table 1:** Driving factor index system for collaborative agglomeration of China’s cultural and tourism industries.

Target System	Primary Power System	Power Subsystem	Power Elements	Target System	Primary Power System	Power Subsystem	Power Elements
**China’s Cultural Industry System**	**The Endogenous Power System**	**Industrial Resource Subsystem**	Number of Museums	**China’s Tourism Industry System**	**The Endogenous Power System**	**Industrial Resource Subsystem**	Number of Scenic Spots
			Number of Public Libraries				Number of Starred Hotels
			Number of Cultural Centers				Number of Travel Agencies
			Number of Art Performance Institutions				Number of Tourism Enterprises
		**Industrial Economy Subsystem**	Proportion of Added Value of Culture and Related Industries in GDP			**Industrial Economy Subsystem**	Total Tourism Income
			Value Added of Cultural Industry				Proportion of Tourism Revenue in GDP
			Growth Rate of Cultural Industry Asset Investment				Value Added of Tourism Industry
			Per Capita Cultural Expenses				Growth Rate of Tourism Industry Asset Investment
		**Human Resource Subsystem**	Number of Employees in Museums			**Human Resource Subsystem**	Number of Employees in Scenic Spots
			Number of Employees in Public Libraries				Number of Employees in Starred Hotels
			Number of Employees in the Cultural Centers				Number of Employees in Travel Agencies
			Number of Employees in Art Performance Groups				Number of Employees in Tourism Enterprise
	**The Regulatory Power System**	**Industrial Policy Subsystem**	Proportion of Cultural Expenses in Financial Expenditure		**The Regulatory Power System**	**Industrial Policy Subsystem**	Proportion of Tourism Expenses in Financial Expenditure
			Financial Investment in Cultural Industry				Financial Investment in Tourism Industry
		**Market Demand Subsystem**	Number of Visitors to the Museums			**Market Demand Subsystem**	Number of Domestic Tourists
			Number of Visitors to the Public Libraries				Number of Inbound Tourists
			Number of Audience of Art Performance Groups				Total Number of People Received in Scenic Spots
		**Innovation Resource Subsystem**	Number of Cultural and Educational Institutions			**Innovation Resource Subsystem**	Number of Tourism Colleges
			Number of Cultural Relics Research Institutions				Number of Students in Tourism Colleges
			Number of Cultural Professionals				Number of Tourism Professionals
		**Social Economic Subsystem**	GDP			**Social Economic Subsystem**	GDP

Note: Data from *Chinese Culture and Tourism Statistical Yearbook, China Tourism Statistical Yearbook, and Chinese Culture and Related Industries Statistical Yearbook from 2008–2019 (S1)*.

### 3.3 Construction of dynamic model for synergistic agglomeration of China’s cultural and tourism industries and analysis of their causal relationship

#### 3.3.1 Construction of collaborative agglomeration power model for culture and tourism industries

**Fig 2:**
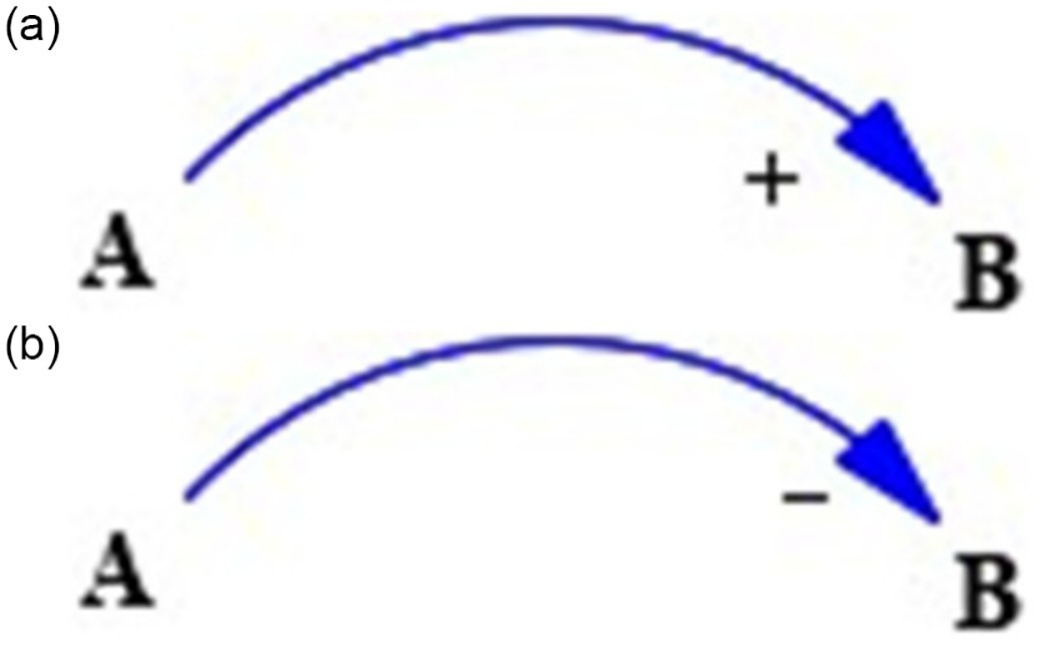
Casual loop diagram of changes between A and B variables with a positive correlation in (a) and a negative correlation in (b).

A system dynamics model (SD Model) is a system engineering method based on systems theory and cybernetics, which reveals the internal laws and feedback mechanism of the system. This method can clearly show the causal relationship, feedback relationship and system behavior characteristics existing in complex systems and has been widely used in the social ecosystem modeling of various environments. In the process of modeling, the high-order nonlinear stochastic partial differential equation describing the system principle can be simplified into a deterministic nonlinear differential equation through the model. Based on the effects of some random and non-deterministic factors in the system, the SD model can be expressed and modeled using test functions. At the same time, when using the SD model for system simulation, adverse factors that cannot be expressed and described mathematically in the system can be benign, and the optimal system dynamics simulation results can be obtained. The modeling process of the SD model follows:

**(1) System analysis**: A system is an organic whole formed by the interconnection of several subsystems and submodules, and the boundaries, structure, and complexity of the system are mainly determined based on the research problem and purpose.
**(2) Analysis of causal relationships among variables**: From a systematic perspective, there is a certain causal relationship between variables, and there is also a certain correlation between the changing trends of variables. In research, positive and negative correlations are generally used to indicate the causal relationship between variables. In the process of system dynamics modeling, it is necessary to first clarify the causal relationship between system variables and represent it through a casual loop diagram (CLD). In the CLD diagram, if the direction of change between A variable and B variable is consistent, which means that the increase (or decrease) of the A variable will cause the increase (or decrease) of the B variable; then, there is a positive correlation between the A variable and B variable, as shown in Fig 2(a). If the increase (or decrease) of Variable A causes the decrease (or increase) of Variable B, a negative correlation exists between Variable A and Variable B, as shown in Fig 2(b).

**(3) Building a system dynamics flow diagram**: A system dynamics flow diagram uses symbols and flow diagram information to describe the interrelationships between variables, and can determine the flow levels, flow rates, and various auxiliary variables in the system for state and rate variables, thereby establishing flow rate equations.
**(4) Model simulation**: After establishing the causal relationship diagram and flow diagram of the system, one must set initial states and values for the variables in the system and set parameters such as simulation start and end times and step sizes. Then, use Vensim software to convert the system simulation model and obtain simulation results.

Based on a qualitative analysis of the dynamic system structure and subsystems of the cultural and tourism industries, this article constructs a collaborative agglomeration dynamic model of the cultural and tourism industries using an SD model. Moreover, this article simulates the development path of collaborative agglomeration of the cultural and tourism industries to explore suitable development paths for collaborative agglomeration of the cultural and tourism industries.

#### 3.3.2 Causal analysis of collaborative agglomeration power system of Chinese culture and tourism industries

**Fig 3:**
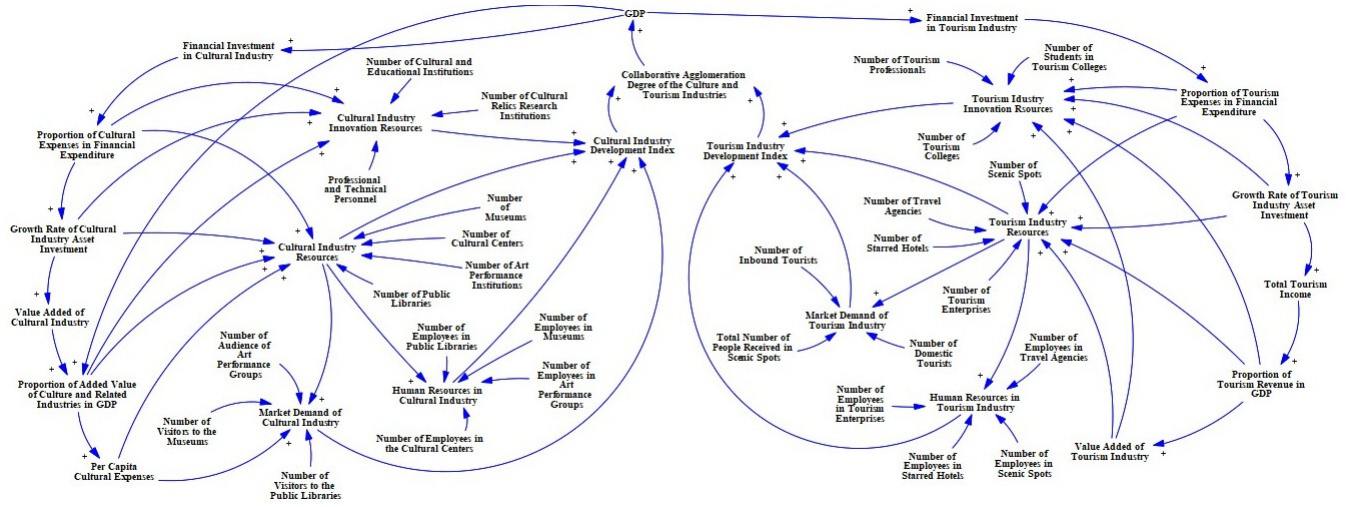
Causal relationship diagram of the collaborative agglomeration power system of Chinese culture and tourism industries.

This article uses an SD model to explore the causal relationship between the driving factors of the collaborative agglomeration of the Chinese culture and tourism industry, laying the foundation for the subsequent establishment of a system flow diagram. The causality diagram is shown in Fig 3, where the symbol “+” represents positive feedback, and the symbol "−" represents negative feedback.

**Fig 4:**
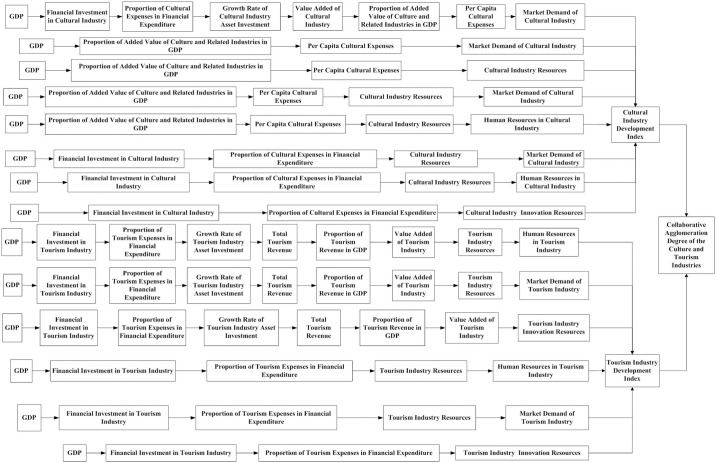
Reason tree of the industrial policy subsystem.

Fig 3 shows how the collaborative agglomeration driving system of China’s cultural and tourism industries includes industrial policy causal loops and industrial economic causal loops:

**(1) Through the analysis of the causal loop of industrial policy (as shown in Fig 4), it can be concluded that**: The government will provide investment, policy guidance, and financial allocation for the cultural and tourism industries. This gives them an open door to intervene and promote the synergistic agglomeration of the cultural and tourism industries. At the same time, government financial support determines the development of innovative resources and industrial resources in the cultural and tourism industries, thereby enhancing the degree of synergy and agglomeration between the cultural and tourism industries. Therefore, within a certain region, the government’s policy support, investment, and allocation of funds for the cultural and tourism industries reflect the government’s emphasis on the cultural and tourism industries. If the government’s attention increases, the support for industries will also expand, thereby accelerating the process of collaborative agglomeration of the cultural and tourism industries. At the same time, the social economic benefits brought by the collaborative agglomeration of the cultural and tourism industries will also increase the government’s attention to both.

**Fig 5:**
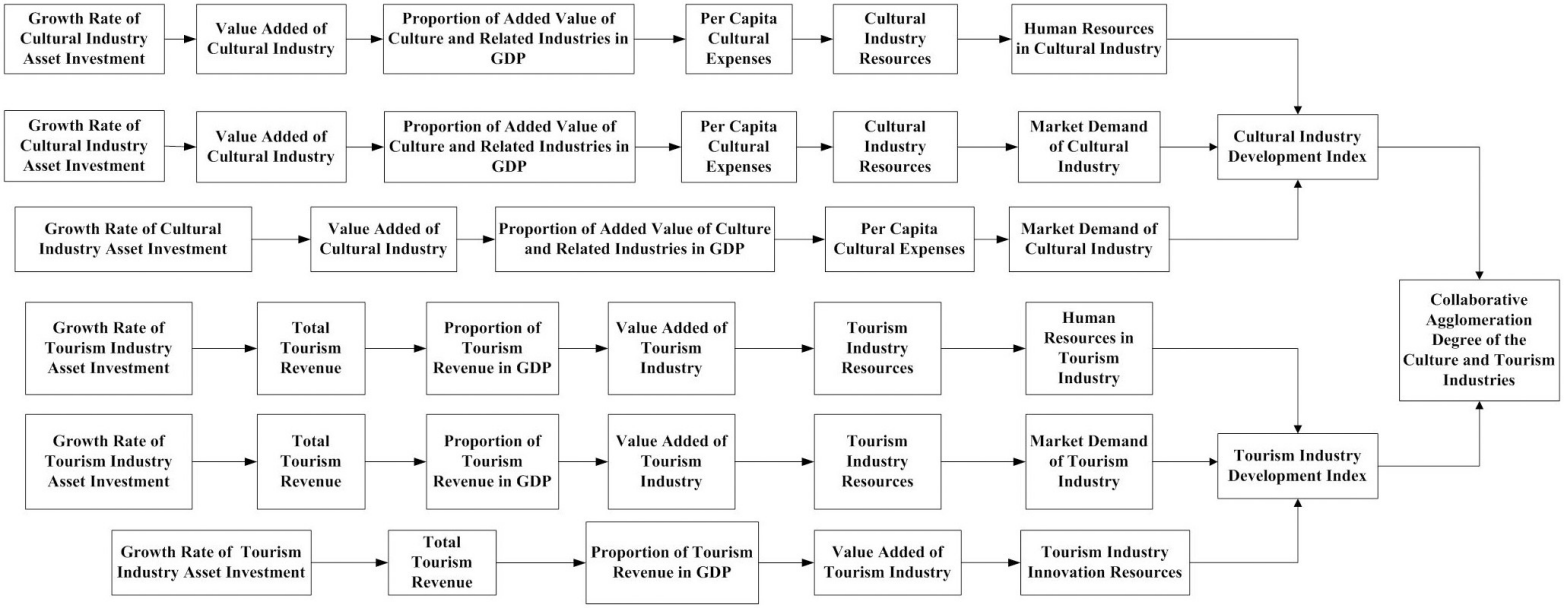
Cause tree of industrial economy subsystem.

**(2) Through the analysis of the causal loop of industrial economy (as shown in Fig 5), it can be concluded that**: The industrial economy subsystem of the collaborative agglomeration power system of cultural and tourism industries mainly emphasizes enhancing the economic strength of the industrial system by increasing the added value of cultural and tourism industries in order to improve the development space of related industries and the economy in the agglomeration area. The cultural industry and tourism industry, as the third industry with a strong correlation in practical development, have a mutually related and promoting relationship in their development. The increase in the added value of the cultural industry will increase the added value of the tertiary industry, leading to the growth of the added value of the tourism industry, thereby forming an economic effect of collaborative agglomeration between the cultural and tourism industries. At the same time, increase in the added value of the cultural and tourism industries will promote the upgrading of the industrial innovation resources and industrial resources, thereby enhancing the supply of human resources and market demand in the industry and further enhancing the degree of collaborative agglomeration between the cultural and tourism industries, forming a sustainable and mutually beneficial development relationship between the cultural industry and economy.

## 3.4 Simulation of the collaborative agglomeration development path of the Chinese culture and tourism industries

**Fig 6:**
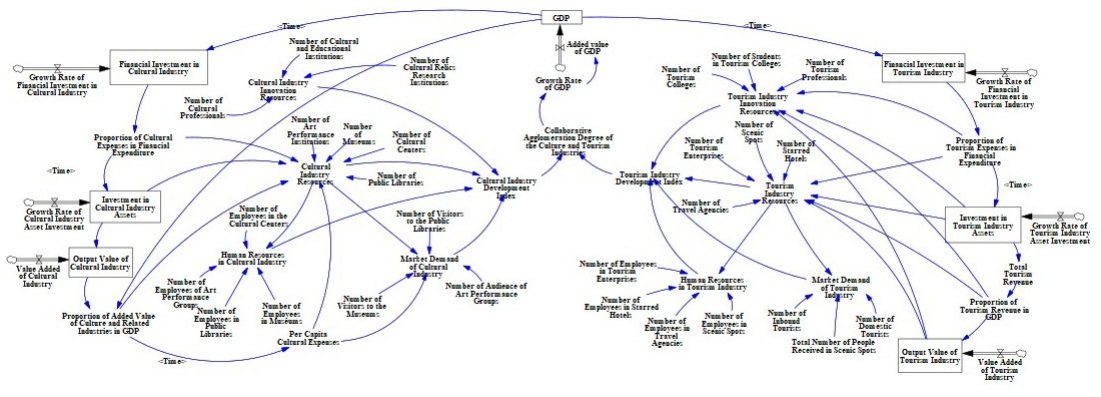
Flow chart of the collaborative agglomeration power system of China’s cultural and tourism industries.

On the basis of clarifying the causal relationship between the collaborative agglomeration of Chinese culture and the tourism industry, this article constructs a dynamic flow chart of its cultural and tourism industries’ collaborative agglomeration system, as shown in Fig 6. This article is based on the causal loop in the dynamic system of the collaborative agglomeration of Chinese culture and tourism industries and analyzes the development path of the collaborative agglomeration of the culture and tourism industries through system simulation.

Prior to system simulation, this article first conducts a system sensitivity test on the collaborative agglomeration power system of China’s cultural and tourism industries. A robust model will not bring about any significant differences due to minor changes in a certain parameter, but rather can ensure that the policy conclusions drawn from model simulation will not change when the parameter changes do not exceed normal values. This article uses historical data method for sensitivity testing. From Fig 6, it can be seen that the six indicators *Financial Investment in Cultural Industry、Investment in Cultural Industry Assets、Output Value of Cultural Industry、Financial Investment in Tourism Industry、Investment in Tourism Industry Assets* and *Output Value of Tourism Industry* are impact variables. Therefore, this article selects the above six indicators for historical testing. The historical data was tested from 2008 to 2019. Related research suggests that the testing error is acceptable when the variation falls between -3% and 3% [26]. The inspection results are shown in Table 2.

**Table 2:** System sensitivity test results.

Index		2008	2009	2010	2011	2012	2013	2014	2015	2016	2017	2018	2019
Year													
**Financial Investment in Cultural Industry**	**True value**	817657	816741	751392	777189	784726	805750	741041	827573	936530	673462	739436	821783
	**Analog value**	817657	813325	746391	767897	781443	800466	737134	820336	931222	670244	730884	813083
	**Error value/%**	0.00	0.42	0.67	1.21	0.42	0.66	0.53	0.87	0.57	0.48	1.17	1.07
**Investment in Cultural Industry Assets**	**True value**	7630	8786	11052	13479	18071	21870	24538	27235	30785	35427	41171	44363
	**Analog value**	7630	8766	11136	13507	18009	21700	24398	27118	80537	35072	40743	43980
	**Error value/%**	0.00	0.22	-0.76	-0.21	0.34	0.78	0.57	0.43	0.81	1.01	1.05	0.87
**Output Value of Cultural Industry**	**True value**	27244	28741	29472	30741	32374	34875	36871	39740	40278	41780	42573	45876
	**Analog value**	27244	28493	29331	30442	32328	34523	36575	335678	40363	41717	42894	45404
	**Error value/%**	0.00	0.87	0.48	0.98	0.14	1.02	0.81	1.21	-0.21	0.15	-0.75	1.04
**Financial Investment in Tourism Industry**	**True value**	3747	4153	4563	4670	5102	5144	7053	10072	12997	15871	13700	10000
	**Analog value**	3747	4143	4540	4649	5107	5163	7012	10001	12880	15705	13766	9955
	**Error value/%**	0.00	0.24	0.51	0.45	-0.10	-0.37	0.59	0.71	0.91	1.06	-0.48	0.45
**Investment in Tourism Industry Assets**	**True value**	11600	11250	12580	19305	22706	26276	30312	34195	39390	45661	51278	57251
	**Analog value**	11600	11371	12725	19340	22690	26143	30060	34450	39190	45285	50765	56943
	**Error value/%**	0.00	-1.06	-1.14	-0.18	0.07	0.51	0.84	-0.74	0.51	0.83	1.01	0.54
**Output Value of Tourism Industry**	**True value**	1.14	1.03	1.57	2.25	2.59	2.95	3.73	4.13	4.69	5.40	5.97	6.63
	**Analog value**	1.14	1.02	1.56	2.23	2.58	2.94	3.70	4.09	4.64	5.37	5.92	6.56
	**Error value/%**	0.00	0.21	0.34	0.45	0.41	0.24	0.70	0.81	0.93	0.42	0.76	0.98

As shown in Table 2, the error values of the main indicators in the collaborative agglomeration power system of China’s cultural and tourism industries fluctuate between -3% and 3%, within an acceptable range of error values. This indicates that the simulation results are basically consistent with the actual situation of system development, and the model has a high degree of structural fit and sensitivity, which can reflect the real situation of the collaborative agglomeration development of China’s cultural and tourism industries.

On the basis of the verification of the sensitivity of the system, this article conducts a system simulation on the causal relationship between the collaborative agglomeration power system of China’s cultural industries and tourism industries in Fig 3. Based on Fig 4, this article divides the development path of the collaborative agglomeration of the cultural and tourism industries into a current situation continuation path, policy support path, economic support path, and policy-economic combination path, and it presents the adjustment to the regulatory variable values of different development paths. Based on the researches of Yang et al. [27], all the regulatory variable values remain unchanged when simulating the status quo continuation path; and all the other regulatory variable values remain unchanged in the policy support path simulation when the values of the industrial policy regulatory variables are adjusted to the maximum; and all the other regulatory variable values remain unchanged in the economic support path simulation when the values of industrial economic regulatory variables are adjusted to the maximum; and all the other regulatory variable values remain unchanged in the policy-economic combination path simulation when all variables of industrial policy and industrial economy are adjusted to their maximum. The specific values are shown in Table 3.

**Table 3:** Simulation parameter settings for the collaborative agglomeration development path of the Chinese culture and tourism industries.

Regulatory variables	Status quo continuation path	Policy support path	Economic support path	Policy-economic combination path
Financial Investment in Cultural Industry	0.131	0.400	0.131	0.400
Investment in Cultural Industry Assets	0.016	0.016	0.131	0.131
Output Value of Cultural Industry	0.011	0.011	0.023	0.023
Financial Investment in Tourism Industry	0.129	0.324	0.129	0.324
Investment in Tourism Industry Assets	0.002	0.002	0.084	0.084
Output Value of Tourism Industry	0.084	0.084	0.242	0.242

**Fig 7:**
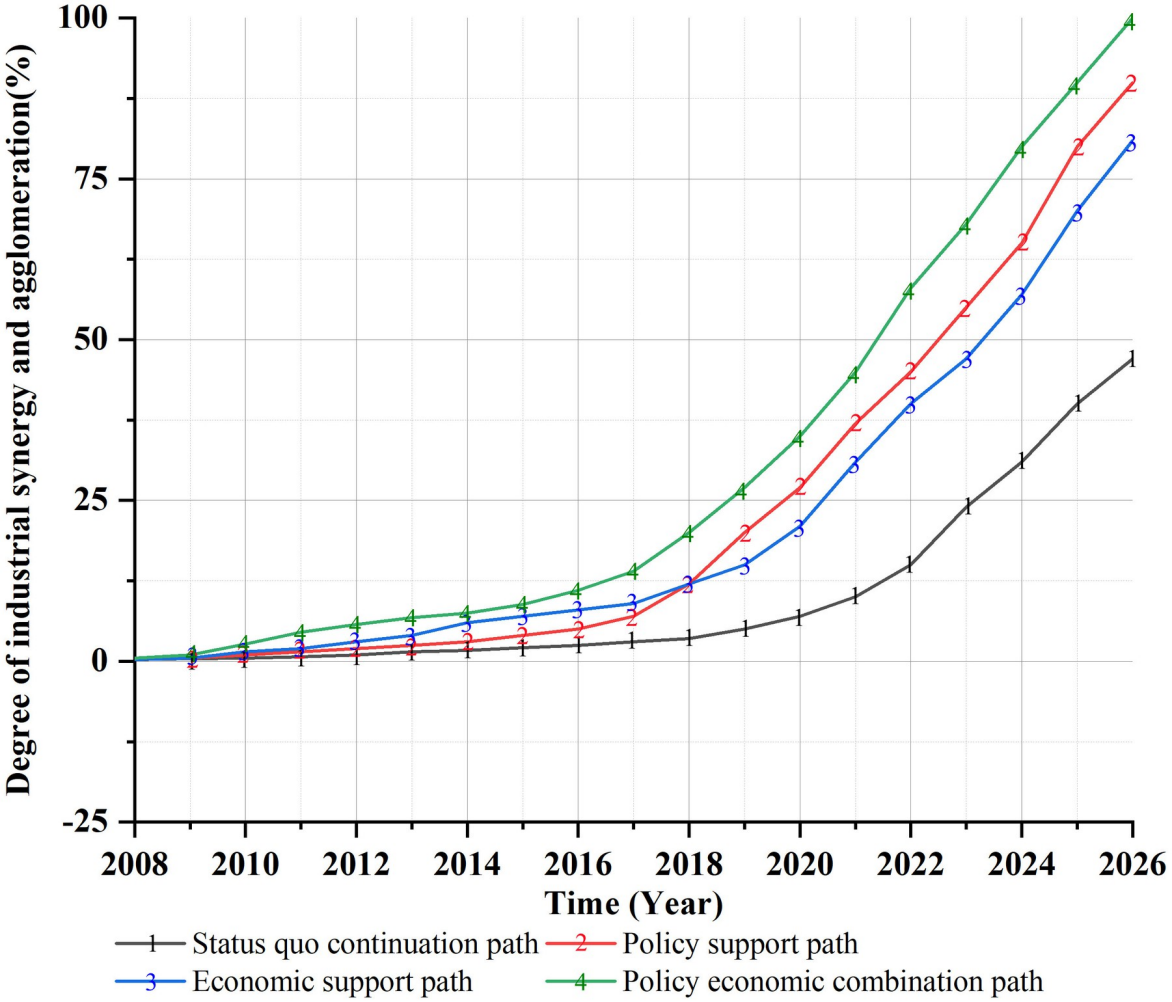
Simulation of the collaborative agglomeration development path of Chinese culture and tourism industries.

By adjusting the parameters of the regulatory variables, the development trend of the degree of synergistic agglomeration of the two industries under the four development paths of collaborative agglomeration of China’s cultural and tourism industries is obtained, as shown in Fig 7:

Fig 7 shows how the policy economic combination path has the best promotion effect on the collaborative agglomeration development of China’s cultural and tourism industries. The impact of the policy support path and economic support path for the collaboration agglomeration of both industries has always been higher than that of the current situation continuation path. This is because policy and economic factors are important influencing factors for the collaborative agglomeration development of China’s cultural and tourism industries.

Under the status quo continuation path, although the degree of collaborative agglomeration between China’s cultural industry and tourism industries has been increasing year by year, its development is slow. From 2008 to 2018, although this path had a certain positive impact on the degree of synergy and agglomeration between China’s cultural and tourism industries, its driving effect was not significant. This is because during this period, industries such as agriculture, manufacturing, and construction contributed more to the Chinese economy and were also key industries for the Chinese government to develop. However, the collaborative relationship between the cultural industry and tourism industry, which developed relatively slowly during this period, is not yet clear. Therefore, the degree of collaborative agglomeration between China’s cultural industry and tourism industry during this period is not good. From 2018 to 2026, the degree of collaborative agglomeration between China’s cultural and tourism industries slightly increased under this path. This is because with the continuous growth of the social economy, both the cultural industry and the tourism industry had developed to a certain extent. At the same time, based on the correlation between the cultural industry and the tourism industry, the collaborative relationship between the two industries became stronger and the degree of collaborative agglomeration continues to gradually increase. However, due to both policy and economic factors being important factors for industrial development, the degree of collaborative agglomeration between China’s cultural and tourism industries slightly increased during this period, but it is still lower than the other three development paths.

Under the policy support path, the promotion effect on the collaborative agglomeration development of Chinese culture and the tourism industry in the early stage was not significant, while the promotion effect in the later stage gradually increased. From 2008 to 2018, the promotion effect of the policy support path (dedicated to the collaborative agglomeration development of China’s cultural and tourism industries) was lower than that of the economic support path and policy economic combination path. This is because during this period, the development level of China’s cultural and tourism industries was relatively low, and the trend of collaborative agglomeration between the two industries was not significant. Additionally, there were few supportive policies for the collaborative agglomeration development of the cultural and tourism industries, and the policy support effect was not significant. From 2018 to 2026, the driving effect of this path on the collaborative agglomeration development of China’s cultural and tourism industries has gradually become significant. This is because during this period, with the rapid development of China’s cultural and tourism industries driven by social economic factors, the collaborative and agglomeration development relationship between industries has become increasingly significant. At the same time, in March 2018, China established the Ministry of Culture and Tourism and introduced policies to promote the collaborative agglomeration development of the two industries. The supporting role of industrial policies in the collaborative agglomeration development of China’s cultural and tourism industries gradually became apparent. Therefore, since 2018, the impact of policy support on the coordinated agglomeration development of China’s cultural and tourism industries has gradually increased and surpassed the economic support path in 2019.

Under the economic support path, the early stage has a certain promoting effect on the degree of collaborative agglomeration development of China’s cultural and tourism industries, and its effect is only second to the policy economic combination path. However, in the later stage, although the effect of this path increases year by year, the growth rate gradually slows down. From 2008 to 2018, the economic support path had a good driving effect on the collaborative agglomeration development of China’s cultural and tourism industries, second only to the policy economic combination path. This is because the decade from 2008 to 2018 represented a period of rapid economic development in China, and industrial economic factors became important influencing factors for the development of industrial synergy and agglomeration. Strengthening economic support for China’s cultural and tourism industries can enhance the degree of collaboration and agglomeration between the two. However, from 2018 to 2026, under the influence of this path, although the degree of collaborative agglomeration between China’s cultural and tourism industries has been increasing year by year, its growth rate has gradually slowed down. This is because since 2018, the Chinese economy has transitioned from a stage of high-speed growth to a stage of high-quality development. The growth rate of the Chinese economy has gradually slowed down, and the impact of industrial economic factors on the collaborative agglomeration development of China’s cultural and tourism industries has gradually weakened. At the same time, with the release of relevant policies on the coordinated agglomeration development of Chinese culture and tourism industries, the impact of industrial policy factors on the collaborative agglomeration development of the two gradually exceeded that of the industrial economic factors. Therefore, after 2018, the impact of the economic support path on the degree of collaborative agglomeration development between the two is lower than that of both the policy support path and the policy-economic combination path.

The policy-economic combination path has always had a better driving effect on the collaborative agglomeration development of China’s cultural and tourism industries—more than the other three development paths, and after 2018, under this path, the growth rate of the collaborative agglomeration degree of China’s cultural and tourism industries also significantly increased. From 2008 to 2018, although the driving effect of this path on the collaborative agglomeration development of China’s cultural and tourism industries had been increasing year by year, its level of collaborative agglomeration was still relatively low. This is because during this period, the development level of China’s cultural and tourism industries was also relatively low, and their collaborative agglomeration trend was not significant. However, due to the fact that industrial policies and economic factors are important influencing factors for the collaborative agglomeration development of China’s cultural and tourism industries, the degree of their collaborative agglomeration development from 2008 to 2018 was still higher than the other three development paths, even though it was at a relatively low level. From 2018 to 2026, with the rapid development of China’s cultural and tourism industries, the trend of collaborative agglomeration between the two gradually became significant. With the assistance of government and social economic policies for the collaborative agglomeration development of the cultural and tourism industries, their growth rate also significantly increased.

## 4. Conclusions and recommendations

This article constructs a dynamic system analysis model of the collaborative agglomeration of the culture and tourism industries using system dynamics. Taking Chinese cultural and tourism industries as research objects, the dynamic system analysis model is designed to simulate the development path of the collaborative agglomeration of the Chinese culture and tourism industries. The main conclusions of this research follows:

(1) From the perspective of industrial policy, the promotion and guidance function of industrial policy elements still needs strengthening in the collaborative development of China’s cultural and tourism industries. Under the influence of the policy support path, the reason why this path could not remarkably promote the collaborative agglomeration development of China’s cultural and tourism industries from 2008 to 2018 was due to the disconnection of industrial policies and the existence of industrial barriers between the two industries. Only by enhancing the integration of cultural and tourism industry policies can we better promote the synergistic agglomeration of cultural and tourism industries. From 2018 to 2026, the integration of government agencies in China’s cultural and tourism industries has overcome the disconnection of development policies between the cultural and tourism industries, and has improved the support effect of industrial policies.
(2) From the perspective of industrial economy, the promotion function of industrial economic factors still needs improving in the collaborative agglomeration and development of China’s cultural and tourism industries. Under the influence of the economic support path, between 2008 and 2018, due to the rapid development of China’s economy, the economic support path played a certain role in promoting the coordinated agglomeration and development of China’s cultural and tourism industries, second only to the policy-economic combination path. From 2018 to 2026, the promotion function of this path has gradually slowed down in the coordinated development of China’s cultural and tourism industries. The reason for this was that the growth of the Chinese economy has gradually slowed down due to its transition from high-speed development to high-quality development. Under the influence of the new economic normality, the driving force of industrial economic factors has also declined in the collaborative agglomeration development of China’s cultural and tourism industries.
(3) From the perspective of the joint effect of industrial policy and economy, the collaborative effect of industrial policies and economic factors is more conducive to promoting the collaborative agglomeration development of China’s cultural and tourism industries. Under the influence of the policy-economic combination path, in the decade from 2008 to 2018, due to the constraints of the underdevelopment of China’s cultural and tourism industries and the imperfect industrial policies, the promotion action of this path had been increasing year by year in the collaborative agglomeration development of the two industries, but it still remained at a relatively low level. From 2018 to 2026, China’s cultural and tourism industries, stimulated by the industrial economy, have been developing rapidly. Meanwhile, benefiting from the sound industrial policies, the promotion action of this path has been significantly enhanced in the collaborative agglomeration development of China’s cultural and tourism industries.

We provide the following recommendations on the basis of the above conclusions: (1) The Chinese government should fully play its guiding role of industrial policies in further coordinating, reforming, and restructuring the government planning agencies for the cultural and tourism industries, in breaking down the administrative barriers in management departments of the cultural and tourism industry, and in improving the forward and backward linkage between the cultural and tourism industries. At the same time, the Chinese government should also be active in shortening the time lag of industrial policies, in improving the coordination between industrial policies, in strengthening policy interaction between different levels of government, in highlighting the pertinence and operability of supporting policies, in exerting the incentive and traction function of industrial policies on the collaborative agglomeration of cultural and tourism industries, and in improving the policy execution efficiency in order to strengthen the continuity and coherence of industrial policies. (2) In the turning stage of China’s economic development, the cultural and tourism industries in China should have also shifted from high-speed development to high-quality development. The development of social economy should promote the optimization and coexistence of the cultural and tourism industries, and should also promote the high-quality integration and development of the two. Simultaneously, fiscal support policies can significantly promote the collaborative agglomeration of the cultural and tourism industries, and can accelerate the collaborative agglomeration development of the two by increasing fiscal expenditure on the cultural and tourism industries. (3) The Chinese government should fully leverage the regulatory and inducing functions in industrial policies and economic factors, and should form a dual spiral development path of synergistic agglomeration of cultural and tourism industries through the combination of such modes as policy guidance and economic support, in order to promote the synergistic agglomeration development of Chinese cultural and tourism industries.

## 5. Limitations and future research

In response to the inevitable trend and practical demands of the collaborative agglomeration development of the cultural and tourism industries, this article analyzes the driving system and development path of collaborative agglomeration based on China’s cultural and tourism industries and their system dynamics. The authors propose countermeasures and suggestions that are conducive to the development of the collaborative agglomeration of the two industries. This article also has certain reference value for the development of cultural and tourism industries in similar regions around the world. However, our study had some limitations. First, based on the principles of the system dynamics models, this article aimed to simulate the development path of cultural and tourism industry collaborative agglomeration on the basis of existing causal loops, which led to ignoring the impact of other variables in the system on the development of the two collaborative agglomeration. Second, due to the availability of research materials, this article did not analyze the structure and driving factors of the collaborative agglomeration system of China’s cultural and tourism industries from the perspective of the entire industry lifecycle and lacked a detailed analysis of each lifecycle stage of industrial collaborative agglomeration.

Future studies can be conducted from the following two aspects: First, during the simulation of the collaborative agglomeration development path of the cultural and tourism industries, continuous supervision and improvement of the variables and parameters of the system model should be carried out to maintain the effectiveness and accuracy of the model. Second, from the perspective of the life cycle of industrial collaborative agglomeration, exploring the dynamic system structure and driving factors of the cultural and tourism industries’ collaborative agglomeration in different stages of industrial development in various regions of China. These new stages will become the focus of subsequent research.

## Supporting information

S1 Dataset Raw data of the driving factors index system for collaborative agglomeration of China’s cultural and tourism industries from 2008 to 2019 (Table 1). (XLSX)

S1 Appendix Explanation of the driving factor index system for collaborative agglomeration of China’s cultural and tourism industries (Table 1). (DOCX)

S2 Appendix The main equations designed in the system dynamica model (Fig 6). (DOC)
