# Supplementary material for: Research on the motivation system and path simulation of collaborative agglomeration of Chinese culture and tourism industries based on system dynamics
Source: PLoS One. 2024 Jan 25;19(1):e0296963. doi: 10.1371/journal.pone.0296963 (PMC10810437; doi:10.1371/journal.pone.0296963)
Supplement: S1 Appendix — (DOCX) [file pone.0296963.s002.docx]

## S2 Appendix 1

Explanation of the driving factor index system for collaborative agglomeration of China's cultural and tourism industries (Table 1).

(1) Number of Museums: The total number of nonprofit institutions in the region that provide the public with access to historical and artistic relics.

(2) Number of Public Libraries: The total number of public cultural facilities open to the public free of charge in the region for collecting, sorting, and preserving literature information and providing inquiry, borrowing, and related services.

(3) Number of Cultural Centers: The total number of institutions that provide free places for cultural activities to the public in the region.

(4) Number of Art Performance Institutions: The total number of all kinds of professional art performance organizations that are sponsored by the regional cultural department or managed by the industry and specialized in performing arts and other activities.

(5) Proportion of Added Value of Culture and Related Industries in GDP: The proportion of the output value created by the cultural industry and the related industrial production activities of all permanent residents in a region in a certain period of time of the regional GDP.

(6) Value Added of Cultural Industry: The added value of unit cultural output value within a unit period.

(7) Growth Rate of Cultural Industry Asset Investment: The added value of investment and financing amounts by the government and enterprises in the cultural industry and its related industries per unit of time.

(8) Per Capita Cultural Expenses: Per capita expenditure of funds for cultural industry in various regions in China.

(9) Number of Employees in Museums: The total number of people who work in museums and receive remuneration.

(10) Number of Employees in Public Libraries: The total number of people who work in public libraries and receive remuneration.

(11) Number of Employees in the Cultural Centers: The total number of people who work in cultural centers and receive remuneration.

(12) Number of Employees in Art Performance Groups: The total number of people who work in art performance groups and receive remuneration.

(13) Proportion of Cultural Expenses in Financial Expenditure: Proportion of funds used for cultural industry in all regions in China in total regional financial funds.

(14) Financial Investment in Cultural Industry: The investment and financing amount of local governments for cultural and related industries.

(15) Number of Visitors to the Museums: The total number of visitors to the museum per unit time.

(16) Number of Visitors to the Public Libraries: The total number of people who borrow and search for information in a public library in a unit of time.

(17) Number of Audience of Art Performance Groups: The total number of people who watch art performances per unit time.

(18) Number of Cultural and Educational Institutions: The total number of institutions engaged in cultural and educational work.

(19) Number of Cultural Relics Research Institutions: The total number of specialized organizations engaged in scientific research on cultural relics.

(20) Number of Cultural Professionals: The total number of personnel with professional skills in cultural inheritance, cultural research, and cultural relic protection.

(21) Number of Scenic Spots: The total number of places with clear geographical boundaries and available for people to visit, stay, and rest.

(22) Number of Starred Hotels: The total number of places providing accommodation, catering, and other services for tourists that meet the evaluation criteria of the China Tourism Administration.

(23) Number of Travel Agencies: The total number of profit-making units that provide tourists with travel, residence, and related tourism services.

(24) Number of Tourism Enterprises: The total number of independent organizations engaged in tourism economic activities.

(25) Total Tourism Income: The total amount of monetary income obtained from the provision of tourism products, shopping products, and other services by the destination country or region to domestic and foreign tourists within a certain period of time.

(26) Proportion of Tourism Revenue in GDP: The proportion of tourists’ total expenses in the process of travel and sightseeing in various regions in China in the GDP.

(27) Value Added of Tourism Industry: The added value of unit tourism output value within a unit period.

(28) Growth Rate of Tourism Industry Asset Investment: The added value of government and enterprise investment and financing in the tourism industry per unit of time.

(29) Number of Employees in Scenic Spots: The total number of people who work in scenic spots and receive remuneration.

(30) Number of Employees in Starred Hotels: The total number of people who work in starred hotels and receive remuneration.

(31) Number of Employees in Travel Agencies: The total number of people who work in travel agencies and receive remuneration.

(32) Number of Employees in Tourism Enterprises: The total number of personnel who establish labor relations with tourism operators and provide tourism services to tourists.

(33) Proportion of Tourism Expenses in Financial Expenditure: Proportion of funds used for tourism industry in all regions in China in total regional financial funds.

(34) Financial Investment in Tourism Industry: The investment and financing amount of local governments for tourism industry.

(35) Number of Domestic Tourists: The number of Chinese mainland residents and foreigners, and overseas Chinese and compatriots from Hong Kong, Macao, and Taiwan who have resided in China for more than one year and stayed in tourist facilities in other places of China for at least one night and at most six months.

(36) Number of Inbound Tourists: The number of foreigners, and overseas Chinese, Hong Kong, Macao, and Taiwan compatriots who come to China to visit, travel, visit relatives, friends, recuperate, investigate, attend meetings, and engage in economic, scientific, technological, cultural, educational, religious and other activities. It does not include the staff of foreign permanent offices in China, such as embassies and consulates, news agencies, enterprise offices, or foreign experts, foreign students, and people who stay on shore for no more than one night.

(37) Total Number of People Received in Scenic Spots: The total number of visitors received by tourist attractions per unit time.

(38) Number of Tourism Colleges: The total number of institutions engaged in tourism education.

(39) Number of Students in Tourism Colleges: The total number of students studying tourism management, hotel management, and other tourism-related majors.

(40) Number of Tourism Professionals: The total number of personnel engaged in professional work such as tourism development and tourism services.

(41)GDP: The total market value of the final product of all production activities in a region per unit time.
