# Supplementary material for: Research on the motivation system and path simulation of collaborative agglomeration of Chinese culture and tourism industries based on system dynamics
Source: PLoS One. 2024 Jan 25;19(1):e0296963. doi: 10.1371/journal.pone.0296963 (PMC10810437; doi:10.1371/journal.pone.0296963)
Supplement: S2 Appendix — (DOC) [file pone.0296963.s003.doc]

## S3 Appendix 2

The main equations designed in the system dynamics model (Fig. 6) are as follows:

(1) INITIAL TIME = 2008

(2) FINAL TIME = 2026

(3) TIME STEP = 1

(4) UNIT OF TIME: Year

(5) Number of Museums = WITH LOOKUP( Time )

(6) Number of Public Libraries = WITH LOOKUP( Time )

(7) Number of Cultural Centers = WITH LOOKUP( Time )

(8) Number of Art Performance Institutions = WITH LOOKUP( Time )

(9) Investment in Cultural Industry Assets = INTEG (Growth Rate of Cultural Industry Asset Investment, 1086.34)

(10) Output Value of Cultural Industry = INTEG (Value Added of Cultural Industry, 7630)

(11) Per Capita Cultural Expenses = WITH LOOKUP( Time )

(12) Number of Employees in Museums = WITH LOOKUP( Time )

(13) Number of Employees in Public Libraries = WITH LOOKUP( Time )

(14) Number of Employees in the Cultural Centers = WITH LOOKUP( Time )

(15) Number of Employees in Art Performance Groups = WITH LOOKUP( Time )

(16) Proportion of Cultural Expenses in Financial Expenditure= WITH LOOKUP( Time )

(17) Financial Investment in Cultural Industry = WITH LOOKUP( Time )

(18) Number of Visitors to the Museums = WITH LOOKUP( Time )

(19) Number of Visitors to the Public Libraries = WITH LOOKUP( Time )

(20) Number of Audience of Art Performance Groups = WITH LOOKUP( Time )

(21) Number of Cultural and Educational Institutions = WITH LOOKUP( Time )

(22) Number of Cultural Relics Research Institutions = WITH LOOKUP( Time )

(23) Number of Cultural Professionals = WITH LOOKUP( Time )

(24) Number of Scenic Spots = WITH LOOKUP( Time )

(25) Number of Starred Hotels = WITH LOOKUP( Time )

(26) Number of Travel Agencies = WITH LOOKUP( Time )

(27) Number of Tourism Enterprises = WITH LOOKUP( Time )

(28) Total Tourism Income = WITH LOOKUP( Time )

(29) Proportion of Tourism Revenue in GDP = Total Tourism Income / GDP

(30) Value Added of Tourism Industry = WITH LOOKUP( Time )

(31) Growth Rate of Tourism Industry Asset Investment = WITH LOOKUP( Time )

(32) Investment in Tourism Industry Assets = INTEG (Growth Rate of Tourism Industry
